# Endothelial Dysfunction in Sex-Specific Disparities in Cardiovascular Diseases: Biological Mechanisms, Diagnostic-Therapeutic Differences, and Translational Medicine Strategies

**DOI:** 10.31083/RCM42766

**Published:** 2025-11-13

**Authors:** Canran Lv, Chu Chen, Cuiyuan Huang, Li Liu, Yunping Sun, Peng Zhu, Zihao Chen, Le Zhang, Jing Zhang, Jian Yang

**Affiliations:** ^1^Department of Cardiology, The First College of Clinical Medical Science, China Three Gorges University & Yichang Central People's Hospital Yichang, 443000 Yichang, Hubei, China; ^2^Hubei Key Laboratory of Ischemic Cardiovascular Disease, 443000 Yichang, Hubei, China; ^3^Hubei Provincial Clinical Research Center for Ischemic Cardiovascular Disease, 443000 Yichang, Hubei, China; ^4^Central Laboratory, The First College of Clinical Medical Science, China Three Gorges University & Yichang Central People's Hospital, 443000 Yichang, Hubei, China; ^5^Key Laboratory of Vascular Aging (HUST), Ministry of Education, Tongji Hospital, Tongji Medical College, Huazhong University of Science and Technology, 430030 Wuhan, Hubei, China

**Keywords:** endothelial dysfunction, cardiovascular disease, sex-specific disparity, sex hormone, translational medicine strategies

## Abstract

Sex-specific disparities in the pathogenesis and outcomes of cardiovascular diseases (CVDs) highlight critical gaps in current clinical paradigms, particularly regarding endothelial dysfunction as a pivotal mediator of such differences. Males have a higher incidence of atherosclerosis-related CVD, while postmenopausal females experience microvascular dysfunction due to estrogen loss and androgen dominance. Estrogen confers cardioprotective effects via nitric oxide (NO)-mediated vasodilation and antioxidant pathways. In contrast, androgens exert dual pathological effects by promoting inflammation and oxidative stress in a concentration-dependent manner. Clinically, men develop obstructive coronary disease, whereas women present with underdiagnosed microvascular ischemia due to sex-neutral thresholds. Sex-specific risks (e.g., smoking/diabetes in women) and treatment disparities persist in CVDs, meaning sex-stratified diagnostics/therapeutics and trial reforms are needed to advance precision cardiology. Unlike traditional reviews that focus on mechanisms, this study aims to link molecular insights with translational strategies by proposing endothelial-targeted therapies, sex-adjusted diagnostic algorithms, and policy-driven trial reforms. By prioritizing the endothelial–sex hormone crosstalk as the nexus of pathophysiology and clinical translation, this synthesis advances precision cardiology beyond conventional symptom-focused paradigms.

## 1. Introduction 

### 1.1 Backgrounds

Cardiovascular disease (CVD) shows significant sex-based epidemiological divergence, with males having a higher lifetime absolute risk than females (68.9% vs. 57.4%, respectively) according to global cohorts like the PURE study [1]. Pathophysiological distinctions are also profound, with over 70% of CVD in females manifesting as coronary microvascular dysfunction (CMVD), driven by endothelial impairment and elevated microcirculatory resistance subsequent to decreased estrogen levels [2]. Conversely, males have a higher susceptibility for plaque rupture—particularly in major vessels like the left anterior descending artery (LAD)—with more than double the risk of females [3].

These sex-specific phenotypes extend to plaque biology. Atherosclerotic plaques in women have distinct morphology with higher lipid density despite a smaller total volume [4, 5], whereas males have a greater risk of CVD associated with elevated non-high-density lipoprotein cholesterol (non-HDL-C) levels (+28% impact vs. females) [1]. Such differences justify the development of sex-adapted risk algorithms and therapeutic strategies [6], while highlighting the need to integrate sex-related variables into CVD management frameworks [7].

### 1.2 The Bridging Role of Endothelial Dysfunction

Endothelial cells play a critical role in maintaining vascular homeostasis by regulating vascular tone, blood flow, and anti-inflammatory responses, thereby supporting cardiovascular health. Endothelial dysfunction is an early hallmark of CVDs such as atherosclerosis. Oxidative stress and inflammation are major drivers of endothelial dysfunction, primarily by reducing the bioavailability of nitric oxide (NO) and increasing the production of reactive oxygen species (ROS), thereby exacerbating endothelial injury [8]. While sex differences in CVD are apparent at the epidemiological level of disease incidence, they also reflect a deeper imbalance in the bidirectional regulation of the sex hormone endothelium axis. Research indicates that sex hormones influence endothelial function through multiple mechanisms, contributing to the pathophysiological differences in CVD observed between women and men [9]. Estrogen mediates endothelial protection primarily through activation of the NO pathway. It does this by upregulating endothelial nitric oxide synthase (eNOS) to enhance vasodilation and vascular integrity. Conversely, androgen imbalance may induce oxidative stress and amplify inflammatory responses. Collectively, these pathogenic factors disrupt endothelial homeostasis, thereby predisposing to cardiovascular pathologies [10, 11]. Therefore, a comprehensive understanding of sex hormone-mediated endothelial regulation and its cardiovascular implications is pivotal for developing precision therapeutics. Potential strategies may involve pharmacological modulation of sex hormone profiles or direct molecular targeting of endothelial dysfunction pathways to restore cardiovascular homeostasis.

### 1.3 Existing Challenges in Clinical Treatment

Epidemiological studies have revealed sex-specific disparities in clinical management pathways for CVDs. For instance, women have significantly lower utilization rates of lipid-lowering agents (16.4% vs. 22.5%, respectively) and antiplatelet therapies (20.9% vs. 34.2%) than men for the secondary prevention of coronary artery disease [12]. Such disparities may be attributed to the female-predominance of microvascular dysfunction pathology, or by the persistent sex imbalance in clinical trial cohorts. A 2024 study reported that male sample bias is prevalent in *in vitro* cardiovascular models, with male-derived samples having a 15% higher cell proliferation rate compared to females [13]. Contemporary guidelines have increasingly emphasized sex-specific clinical protocols (e.g., prioritizing the assessment of coronary flow reserve (CFR) in women) to reduce sex-based disparities through precision medicine. However, widespread implementation requires holistic advances across policy support, resource optimization, and multidisciplinary collaboration, ultimately establishing a closed-loop system to translate evidence-based guidelines into sex-specific clinical practice.

## 2. Biological Mechanisms​

### 2.1 Protective Effects of Estrogens

The biological mechanisms underlying sex hormone-mediated endothelial regulation represent a multifaceted and critical domain in biomedical research. In particular, the cardioprotective role of estrogen has been extensively studied, with a particular focus on its endothelial effects. Accumulating evidence shows that estrogen confers cardiovascular protection via diverse molecular mechanisms. Primarily, it activates intracellular signaling pathways through receptors including estrogen receptor-α (ERα), estrogen receptor-β (ERβ), and G protein-coupled receptor 30 (GPER-1, previously named GPR30). This mediates genomic transcription and rapid non-genomic responses, thereby modulating endothelial cell activity through potentiation of the NO pathway and attenuation of oxidative stress [14]. Activation of these receptors promotes NO production, which is critical for vasodilation and vascular health [15]. Estrogen-bound ERα/ERβ rapidly activate tyrosine kinase Src, extracellular signal-regulated kinase 1/2 (ERK1/2), phosphoinositide 3-kinase (PI3K), and protein kinase B (Akt), leading to eNOS Ser1177 phosphorylation and NO release within minutes [16]. Estradiol (E2), the predominant endogenous estrogen in humans, binds to GPER-1 and induces eNOS phosphorylation via sequential activation of the Src, epidermal growth factor receptor (EGFR), and PI3K signaling pathways [17]. Beyond the rapid activation of eNOS, estrogen binding to ERα/ERβ/GPER-1 also upregulates eNOS expression, thus promoting sustained vascular relaxation. Park *et al*. [18] found that GPER-1-mediated eNOS induction is dependent on the transcription factor Kruppel-like factor 2 (KLF2). In addition to its gasotransmitter-mediated regulation of vascular tone, estrogen also reduces endothelin-1 (ET-1) biosynthesis by downregulating the expression of preproET-1 and activity of endothelin-converting enzyme (ECE) activity, thereby suppressing ET-1-mediated vasoconstriction [19].

Estrogen also protects the cardiovascular system by modulating oxidative stress, a key pathological mechanism underlying atherosclerosis and other cardiovascular disorders. It achieves this through dual mechanisms: reducing ROS generation and increasing antioxidant enzyme activity to preserve cardiovascular homeostasis [20, 21].

**Fig. 1.**
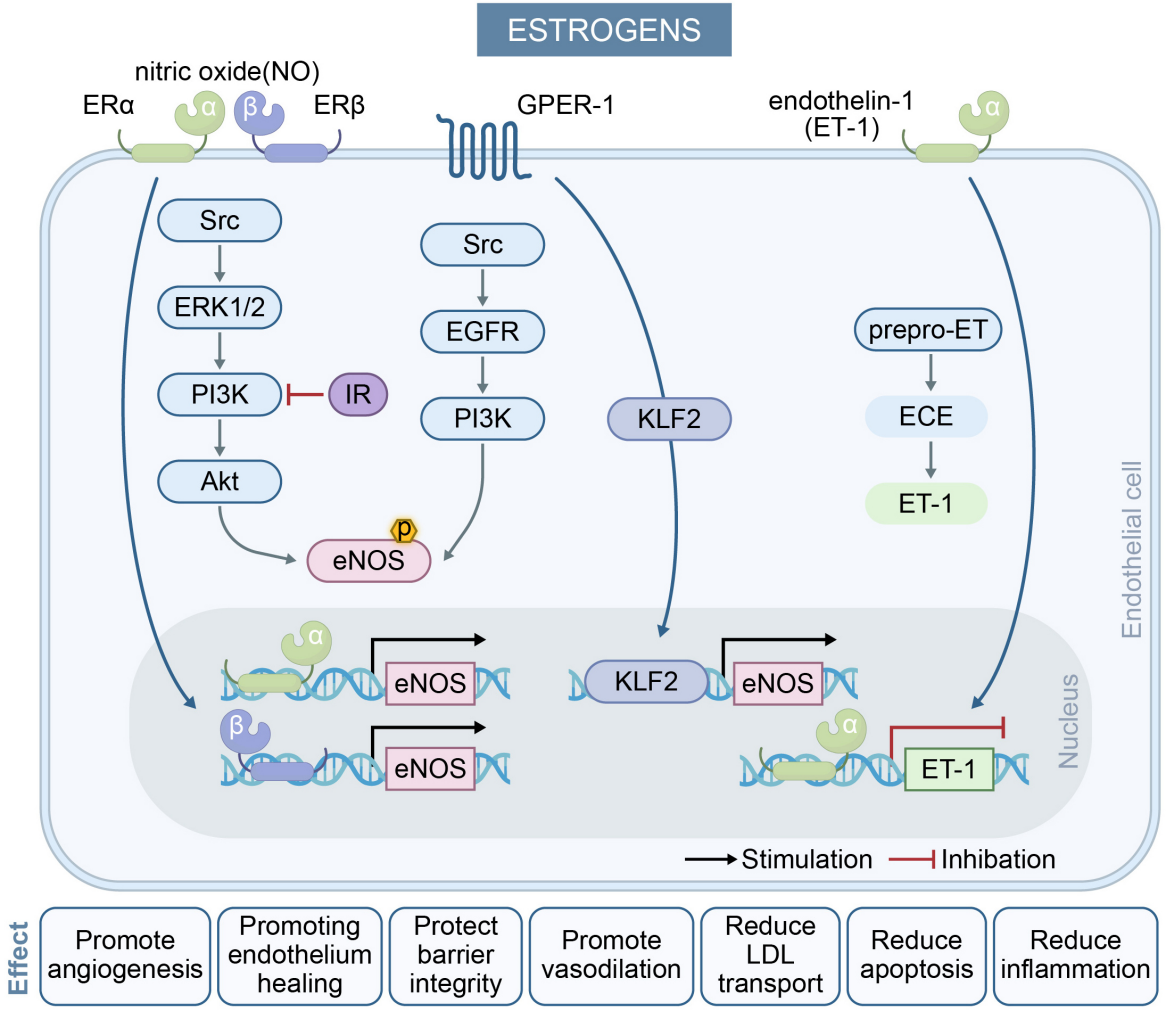
**The effect of estrogens on endothelial cells and their key mechanisms in regulating endothelium-dependent vasodilation**. Estrogens activate ERα, ERβ, and GPER-1 receptors on endothelial cells, thereby enhancing angiogenesis, barrier integrity and vasodilation, and suppressing inflammation/apoptosis to sustain vascular homeostasis. In the NO pathway, ​​estrogens​​ activate eNOS within minutes by inducing two signaling pathways (blue arrows) and enhancing eNOS transcription (black arrows), resulting in long-term induction of NO levels. In postmenopausal women, insulin resistance (IR) contributes to reduced NO and impaired endothelium-dependent vasodilation. In the ET-1 pathway, ​​estrogens​​ reduce ET-1 through ERα binding. And they decrease transcription of its converting enzyme ECE (red inhibitory arrow). ERα, estrogen receptor-α; ERβ, estrogen receptor-β; GPER-1, G protein-coupled receptor 30; NO, nitric oxide; eNOS, endothelial nitric oxide synthase; ECE, endothelin-converting enzyme; ERK1/2, extracellular signal-regulated kinase 1/2; PI3K, phosphoinositide 3-kinase; Akt, protein kinase B; EGFR, epidermal growth factor receptor; KLF2, Kruppel-like factor 2; LDL, low-density lipoprotein.

In summary, estrogen improves vascular function by modulating the expression and activity of eNOS, thereby promoting NO production. This process is critical for inducing vasodilation, suppressing platelet aggregation, and attenuating inflammatory responses. Additionally, estrogen maintains endothelial integrity and functionality through its regulatory effects on endothelial cell proliferation and apoptosis [15]. Fig. 1 illustrates the regulation of endothelial cell function by estrogen.

### 2.2 The Dual Effect of Androgens

Androgens exert transcriptional regulatory effects through binding to nuclear androgen receptors (ARs) and G protein-coupled receptors, with subsequent effects on multiple target organs including the cardiovascular system. ARs are ubiquitously expressed in vascular endothelial and smooth muscle cells, where they play critical roles in modulating cardiovascular functions such as NO release, calcium ion mobilization, vascular cell apoptosis, hypertrophy, calcification, senescence, and ROS generation [22, 23]. Androgens have dual effects in the cardiovascular system, with outcomes that can manifest differently depending on the sex-specific biological contexts and physiological states. Physiological concentrations of testosterone (T) rapidly enhance NO production in endothelial cells via a non-genomic pathway that is independent of T-to-E2 conversion [24, 25]. Furthermore, T activates the protein kinase C (PKC) and mitogen-activated protein kinase (MAPK) pathways to amplify NO synthesis [26]. Goglia *et al*. [27] reported that physiological levels of T significantly elevate NO generation and induce the phosphorylation of endothelial eNOS through the PI3K/Akt signalling cascade, thereby increasing NO bioavailability and improving vasodilatory function. Notably, the local conversion of T to E2 by aromatase (CYP19A1) can also induce eNOS activation [28].

Conversely, androgens (e.g., T and dihydrotestosterone) were found to enhance tumor necrosis factor-α (TNF-α)-induced inflammatory responses in human umbilical vein endothelial cells (HUVECs) derived from both sexes, whereas estrogen did not show analogous pro-inflammatory activity [29]. This observation indicates that androgens may contextually promote inflammatory pathways, thereby amplifying cardiovascular risk by increasing endothelial inflammation. At pathological concentrations, androgens mediate deleterious effects via dysregulation of signaling pathways [30]. Clinical data have shown that women with polycystic ovary syndrome (PCOS) exhibit a 50% impairment in endothelium-dependent vasodilation compared to healthy controls, correlating with hyperandrogenemia-induced endothelial dysfunction [31]. Similar to estrogen, androgens have been shown to modulate the expression of ET-1. A cross-sectional study demonstrated a positive correlation between plasma T levels and ET-1, suggesting that elevated androgen exposure mediates vasoconstriction [32].

Sex chromosome differences could also play an important role in the development of CVD, with inherently unequal effects of sex chromosome genes influencing sex-specific control of this disease. For example, the second X chromosome may have an adverse effect in females that is not present in males. This interplay of sex chromosome and sex hormone mechanisms may manifest as mutually antagonistic effects in certain diseases [33].

**Fig. 2.**
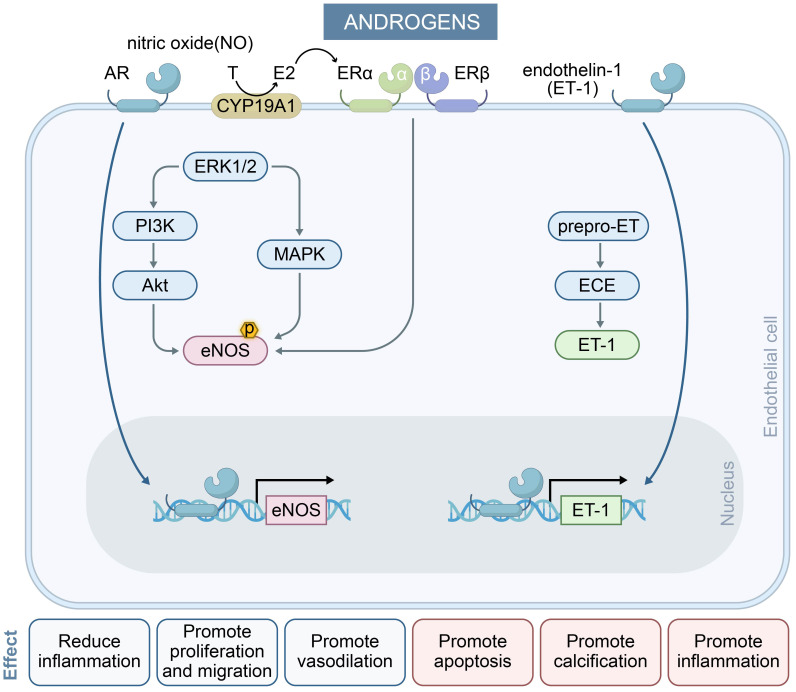
**The effect of androgens on endothelial cells and their key mechanisms in regulating endothelium-dependent vasodilation**. Androgens exert dual effects on endothelial cells via AR signaling. They stimulate pro-inflammatory, pro-apoptotic, and calcification pathways (right) while promoting vasodilation, proliferation, and anti-inflammatory responses (left), reflecting context-dependent vascular modulation. In the NO pathway, physiological concentrations of ​​androgens​​ activate eNOS within minutes by inducing two signaling pathways (blue arrows) and enhancing eNOS transcription (black arrows), resulting in long-term induction of NO levels. In the ET-1 pathway, elevated androgen levels induce ET-1 transcription (black arrows) and mediate vasoconstriction. MAPK, mitogen-activated protein kinase; AR, androgen receptor.

In summary, the dual nature of androgens stems from the concentration-dependent activation of androgen receptor signaling pathways, with physiological concentrations predominantly activating non-genomic rapid pathways, while elevated concentrations drive genomic pathways that induce chronic damage. These findings provide novel insights into the sex disparities observed in CVD and also suggest potential targets for clinical intervention. The regulation of endothelial cell function by androgen is shown in Fig. 2.

### 2.3 Transitions in Female Menopause

The menopausal transition represents a critical physiological juncture in women, characterized by profound alterations in sex hormone profiles. Emerging evidence indicates these endocrine shifts directly modulate cardiometabolic risk profiles, with the depletion of estrogen correlating with accelerated vascular aging and endothelial dysfunction [34].

The decline in endothelial function during this period may stem from the dual effects of estrogen depletion and relative androgen elevation. Progressive deterioration of endothelial function with menopausal progression may be attributable primarily to the loss of ovarian estradiol [35]. In addition, menopause is frequently accompanied by IR, a metabolic stress condition that further aggravates endothelial injury. The decline in estrogen level, combined with IR during menopause, leads to diminished NO synthesis and impaired endothelium-dependent vasodilation, serving as a foundational trigger for atherogenesis [24, 36]. The mechanism by which IR exerts its effects is shown in Fig. 1. Concurrently, estrogen deficiency upregulates inflammatory mediators (e.g., IL-6, TNF-α) and oxidative stress markers (e.g., ROS), thereby accelerating endothelial injury through activation of the NF-κB pathway and mitochondrial dysfunction [37]. IR induces a pro-inflammatory, pro-oxidative vascular milieu, further aggravating these pathological conditions. Conversely, menopausal women may experience relative androgen elevation (e.g., androgen-to-estrogen ratio imbalance) due to ovarian hypofunction, which exerts complex effects on endothelial function. Specifically, postmenopausal decline in the level of sex hormone-binding globulin (SHBG) enhances free androgen bioavailability, thereby amplifying the detrimental effects of hyperandrogenism on endothelial homeostasis through mechanisms including ROS overproduction and pro-inflammatory cytokine dysregulation [38]. This effect results in microvascular dysfunction, which in turn further exacerbates IR. Hormonal imbalance and metabolic stress act in concert, synergistically amplifying endothelial dysfunction and increasing cardiovascular risk.

Consequently, research on hormone replacement therapy (HRT) in postmenopausal women continues to explore the strategy of restoring estrogen’s cardiovascular protective effects. However, outcomes for HRT in the prevention of CVD are inconsistent, and are likely to be influenced by factors including estradiol type, dosage, formulation, administration route, timing, and treatment duration. Additional variables such as hormonal milieu, vascular health status, and menopause-related dysfunction could also modulate HRT efficacy [39], necessitating individualized treatment plans.

### 2.4 Core Mechanisms of Endothelial Dysfunction and Potential Research Directions

NOD-like receptor pyrin domain-containing 3 (NLRP3) is a cytosolic multi-protein complex that induces inflammation. The ROS-NLRP3 pathway functions as the central driver of endothelial dysfunction (ED). ROS directly activate assembly of the NLRP3 inflammasome, triggering caspase-1-mediated cleavage and release of IL-1β/IL-18, which then induces endothelial inflammation and gasdermin D (GSDMD)-dependent pyroptosis. This endothelial insult amplifies ROS generation, establishing a self-perpetuating “ROS-NLRP3-inflammation-ED” vicious cycle. Critically, this pathogenic axis underpins ED across a spectrum of cardiovascular conditions, including metabolic syndrome (e.g., IR), ischemia-reperfusion injury (IRI), and vascular aging [40].

While direct evidence linking sex hormones to ED via ROS-NLRP3 signaling remains limited, studies with non-endothelial models have demonstrated their regulatory control over this pathway [41, 42]. Given the established role of ROS-NLRP3 crosstalk as a core driver of endothelial injury, future investigations should investigate the mechanism of sex-specific modulation of this axis in cardiovascular pathogenesis.

## 3. Sex-Specific Clinical Presentations ​

### 3.1 Sex Differences in CVD

CVDs exhibit marked sex-related differences in their clinical expression. Women with acute coronary syndrome (ACS) present more often with atypical symptoms (e.g., fatigue/dyspnea) and non-obstructive coronary artery disease compared to men, while younger women have higher rates of ACS without detectable lesions [43, 44]. Women with heart failure (HF) predominantly develop preserved ejection fraction phenotypes (HFpEF), in contrast to the predominance of ischemic cardiomyopathy in males [45]. Social determinants further contribute to sex-based disparities, with women exhibiting higher vulnerability to major life stressors and depression prior to cardiovascular events [46].

These clinical distinctions reflect underlying pathophysiological differences, with cardiomyocytes in females showing enhanced resistance to oxidative stress, mediated partially through the antioxidant properties of estrogen [47]. Importantly, biological sex modulates disease expression across all cardiomyopathy subtypes [48], confirming it is a fundamental variable in CVD manifestations.

### 3.2 Differences in Endothelial Function Testing

CVDs demonstrate marked sex differences in clinical presentation, with assessment of endothelial function serving as a key tool for uncovering these disparities. Studies have shown that endothelial function is a sensitive indicator of cardiovascular health and can also predict the development of atherosclerosis [10]. Premenopausal women generally have a lower risk of CVD compared to men, which is largely attributed to the protective effects of estrogen on endothelial function [49].

Further, sex differences are also evident in the number and activity of endothelial progenitor cells (EPCs). Premenopausal women have significantly higher circulating EPC counts and activity compared to men, likely due to the regulatory effects of estrogen. Women with hypertriglyceridemia, also demonstrate superior recovery of endothelial function compared to men with this condition, adding support to the existence of sex differences in endothelial function [49].

Additionally, women with chronic kidney disease (CKD) tend to exhibit poorer arterial wave reflection and microvascular function than men with CKD. This may partly explain the higher risks of CVD and mortality observed in women with end-stage renal disease [50]. A study has also revealed that women exhibit distinct protein expression profiles in CVD biomarkers compared to men. These differences may be associated with biological pathways involving inflammation, lipid metabolism, fibrosis, and platelet homeostasis [51].

In atherosclerotic CVD, sex differences are evident not only in the clinical presentation and pathogenesis, but also in the sex-specific regulation of endothelial function. Estrogen is believed to have cardioprotective effects in women, whereas androgens may have a detrimental impact on cardiovascular health in men [52]. The mechanisms underlying these sex differences are likely related to the interactions between sex hormones and their receptors [53].

In summary, the assessment of endothelial function plays a crucial role in uncovering sex differences in CVDs. In-depth investigation of the underlying mechanisms can increase our understanding of the sex-specific nature of cardiovascular conditions, while offering new perspectives and strategies for personalized treatment.

## 4. Sex-Specific Risk Factors ​

Traditional cardiovascular risk factors, such as hypertension, diabetes, smoking, and hyperlipidemia, may have differing impacts on men and women. The risk of CVD in women has been shown to accelerate after menopause due to changes in vascular function and lipid profiles. Moreover, pregnancy offers a unique window to screen otherwise healthy women who may be at increased risk of future CVD [54]. Sex-specific risk factors for carotid intima-media thickness and plaque progression also differ in high-risk populations. In women, total cholesterol and diabetes are significantly associated with the development of new plaques, whereas, smoking and elevated triglyceride levels show stronger associations in men [55].

In addition to traditional risk factors, women are also affected by unique sex-specific risk factors such as adverse pregnancy outcomes, early menopause, and chronic autoimmune inflammatory diseases. Psychosocial risk factors including stress, depression and social determinants of health may also exert a disproportionately negative impact on women [56]. These sex-specific risk factors should be integrated into assessments of cardiovascular risk to enable the development of personalized preventive strategies [57].

Sex-specific proteomic profiles have also been shown to increase the predictive accuracy of cardiovascular risk. The incorporation of sex-specific protein concentrations can significantly improve the discriminatory power of risk prediction models for 10-year major adverse cardiovascular events (MACE) [58]. Moreover, sex-specific biomarkers have demonstrated subtle differences in their prediction of HF, although the management of overall risk factors remains equally important for both sexes [59].

In conclusion, sex-specific risk factors play critical roles in the diagnosis, risk assessment and management of CVD. Understanding these differences can help clinicians develop more effective prevention and treatment strategies, ultimately improving cardiovascular health in both women and men [60].

## 5. Sex-Specific Differences in Treatment Response and Prognosis

Sex-based pathophysiological differences can significantly modulate therapeutic efficacy and outcomes in CVD. Women typically present at older ages with higher post-event mortality, partly because their elevated platelet reactivity attenuates the effects of antiplatelet therapy [61, 62]. These distinctions, which span the initial presentation, pathophysiology, and clinical trajectories, necessitate sex-adapted diagnostic and therapeutic guidelines to optimize the outcomes of female patients [63]. However, the underrepresentation of women in clinical trials limits the applicability of evidence-based therapy, underscoring the importance of integrating sex-specific pharmacokinetic/pharmacodynamic profiles into drug development frameworks [64].

### 5.1 Hormone Replacement Therapy

HRT has been a topic of considerable interest in the context of CVD, particularly the study of how sex differences influence treatment response and outcomes. The risk of CVD has been shown to increase significantly in women after menopause, following a trend that is closely linked to the decline in estrogen level [65]. However, the role of HRT in the prevention and treatment of CVD remains highly controversial within the scientific community.

An initial observational study suggested that HRT might have a protective effect against CVD. However, results from randomized controlled trials (RCTs) have been inconsistent [66]. For example, large clinical trials such as the Heart and Estrogen/progestin Replacement Study (HERS) and the Women’ s Health Initiative (WHI) found that HRT did not confer significant cardiovascular protection in women with existing atherosclerosis and may even increase the risk of certain cardiovascular events [67]. Moreover, the effects of HRT may depend on various factors, including the type and formulation of hormones used, timing of initiation, dosage, route of administration, and the patient’s age and pre-existing cardiovascular status [39, 68]. Some studies have suggested that initiating HRT during the early postmenopausal period may confer cardiovascular benefits, whereas delayed initiation may be ineffective or even harmful [69]. Therefore, individualized HRT regimens are particularly important to optimize outcomes and minimize risks.

The effects of HRT also vary by sex. For example, in the context of growth hormone replacement therapy (GHRT), men and women show different degrees of improvement in cardiovascular risk factors, with men generally showing more pronounced benefits [70]. This highlights the importance of taking into account sex differences when considering the use of HRT in clinical practice.

Finally, although HRT may confer cardiovascular benefits in certain contexts, its potential risks cannot be overlooked. Current evidence suggests that HRT should not be used as a primary strategy for CVD prevention, but should instead be considered cautiously based on individual patient profiles [71]. Further research is needed to fully elucidate the specific mechanisms of HRT in diverse populations, with the aim of providing clearer guidance for clinical practice [72].

### 5.2 Differences in Drug Response

Significant sex-based differences have been demonstrated in the pharmacokinetics and pharmacodynamics of cardiovascular drugs, with possible impacts on therapeutic efficacy and safety [73]. First, women differ from men in the absorption, distribution, metabolism, and excretion of cardiovascular drugs. These variations may increase the likelihood of adverse drug reactions in women. For example, women often face a higher risk of drug toxicity, partly due to smaller kidney size and lower glomerular filtration rates. In addition, the inhibitory effects of estrogen on the central sympathetic nervous system and the renin-angiotensin system can further influence drug responses [74]. Second, sex differences are also evident in the clinical efficacy of medications. For example, women with HF often do not achieve the same therapeutic benefits from pharmacologic treatment as men. This may be attributed to the underrepresentation of women in clinical trials and the lack of sex-specific dosing recommendations. Women are also more prone to serious adverse reactions during HF treatment, suggesting that lower drug doses may be more appropriate for female patients [75].

Compared to men, women also exhibit distinct responses to medication for the treatment of coronary artery disease (CAD). They often experience different efficacy and adverse effect profiles when using antiplatelet and anticoagulant therapies, possibly due to anatomical and physiological characteristics, such as smaller heart size, higher heart rate, shorter cardiac cycle, and longer QT interval [76].

### 5.3 Differences in Prognosis

Emerging evidence highlights sex-specific disparities in CVD prognosis, with women facing higher risks for certain clinical endpoints compared to men. For example, the treatment of ACS presents sex-specific challenges, with female patients having higher in-hospital mortality and an increased risk of MACE within one year compared to their male counterparts [77].

Women may also require different optimal dosages of certain medications for the treatment of HF, and may derive different levels of benefit from device-based therapies compared to men [78]. However, the underrepresentation of women in RCTs for HF limits our ability to accurately assess the sex-specific efficacy and safety of treatments [79]. In dilated cardiomyopathy (DCM), male patients have a higher risk of all-cause mortality compared to females, which may be related to a greater severity of ventricular dysfunction and a higher burden of myocardial scarring [80]. Sex differences are also pronounced in CAD, and women with stable CAD are more likely to present with multiple comorbidities, have a higher prevalence of psychosocial risk factors, and exhibit atypical symptoms. These factors may contribute to sex-related differences in the treatment of both stable angina and ACS. Comorbidity-driven prognostic challenges therefore require secondary prevention protocols that are specific for men or women [81].

## 6. Future Research Directions

### 6.1 Clinical Translation of Sex-Specific Biomarkers

Despite mandates from institutions such as the NIH requiring the inclusion of sex-based analyses, implementation remains inadequate. More than half of clinical trials fail to adequately report sex-specific data, thereby undermining the development of evidence-based precision medicine and limiting informed clinical decision-making [82]. CVD research has shown that sex differences not only influence disease manifestations but also have a significant impact on the interpretation and clinical application of key biomarkers.

Sex differences significantly influence the expression and clinical application of cardiovascular biomarkers. Baseline levels of markers related to inflammation, lipid metabolism, and myocardial injury, such as high-sensitivity cardiac troponin T (hs-cTnT), are known to differ between men and women. The use of sex-specific thresholds can improve diagnostic accuracy for acute myocardial infarction (AMI). In healthy populations, women generally have lower hs-cTnT levels than men. The application of sex-specific reference values (14 ng/L for men and 11 ng/L for women) can increase the diagnostic accuracy of hs-cTnT to as high as 91% [83]. In the early diagnosis of ACS, the 99th percentile levels of myocardial injury biomarkers are significantly influenced by sex. While modern assays with high-sensitivity can detect even minor myocardial damage, the use of sex-specific thresholds is essential to take into account physiological differences, thus avoiding diagnostic misclassification [84]. These findings highlight the importance of developing sex-specific reference frameworks for cardiovascular biomarkers, allowing optimization of precision medicine. Such approaches are particularly valuable in emergency settings, where rapid triage and early diagnosis of ACS rely heavily on the accurate interpretation of biomarkers [51].

### 6.2 Policy-Driven Therapeutic Optimization ​​​

Multiple challenges and opportunities exist to promote sex balance in clinical trials. In studies of diseases with significant sex differences (e.g., depression), women often respond differently to treatment than men. However, many trials continue to ignore sex factors, thereby undermining the generalisability of their results [85].

Circulating extracellular vesicles (EVs) are heterogeneous, membrane-bound structures derived from diverse cellular sources, including endothelial cells. EVs have recently emerged as valuable tools for both prognostication and therapeutic intervention in multiple pathological conditions, including CVDs. Their role in sex-dimorphic endothelial dysfunction offers novel translational strategies, whereby EVs are first generated from sex-stratified sources (e.g., female adipose-derived mesenchymal stromal cells) enriched with protective miRNAs (e.g., miR-200, miR-126) that target endothelial pathways. Next, sex-specific exercise regimens are leveraged to modulate the release of endogenous EV profiles, thus boosting cardioprotective EV subpopulations (e.g., CD62E+ large EVs in women) as both therapeutic agents and biomarkers. Finally, endothelial EV signatures (e.g., endoglin+ MPs, miR-320 cargo) are used diagnostically to identify high-risk cohorts for tailored EV therapies. This integrated approach combines precise EV engineering, lifestyle-induced EV modulation, and EV-guided patient stratification to enable targeted mitigation of sex-divergent endothelial damage mechanisms in CVD [86].

Policymakers, research institutions and healthcare providers must work collaboratively to design new therapeutic strategies for CVDs that take into account sex differences. Moreover, Electronic Health Records (EHR) should be integrated with embedded clinical decision support (CDS) systems to trigger sex-specific therapy alerts, thus ensuring real-world translation of trial data.

## 7. Conclusions

Although both sex-dependent and independent mechanisms intricately regulate cardiovascular endothelial physiology, their roles in disease development remain incompletely understood. NO-mediated protection by estrogen and the duality of androgenactin are well recognized, but further investigation of cardiovascular region-specific sex dimorphism in endothelial responses is needed. Persistent gaps continue to impede translational progress in this field, including nonreporting of the sex origin of cells used in research models, and the male dominance of clinical trials. Future studies should endeavour to integrate sex-stratified cardiovascular endothelial analyses across distinct anatomical sites, as well as mandating the analysis and reporting of sex-disaggregated data. Such approaches should help to elucidate context-dependent mechanisms and drive precision therapies that take into account both hormonal profiles and CVDs heterogeneity.
